# Participation in mental healthcare: a qualitative meta-synthesis

**DOI:** 10.1186/s13033-017-0174-y

**Published:** 2017-11-07

**Authors:** Norman J. Stomski, Paul Morrison

**Affiliations:** School of Health Professions, Murdoch University, ECL 2049 90 South St, Murdoch, WA 6150 Australia

**Keywords:** Participation, Mental health, Meta-synthesis

## Abstract

**Background:**

Facilitation of service user participation in the co-production of mental healthcare planning and service delivery is an integral component of contemporary mental health policy and clinical guidelines. However, many service users continue to experience exclusion from the planning of their care. This review synthesizes qualitative research about participation in mental healthcare and articulates essential processes that enable service user participation in mental health care.

**Methods:**

Electronic databases were systematically searched. Studies were included if they were peer reviewed qualitative studies, published between 2000 and 2015, examining participation in mental health care. The Critical Appraisal Skills Program checklist was used to assess the quality of each included study. Constant comparison was used to identify similar constructs across several studies, which were then abstracted into thematic constructs.

**Results:**

The synthesis resulted in the identification of six principal themes, which articulate key processes that facilitate service user participation in mental healthcare. These themes included: exercising influence; tokenism; sharing knowledge; lacking capacity; respect; and empathy.

**Conclusions:**

This meta-synthesis demonstrates that service user participation in mental healthcare remains a policy aspiration, which generally has not been translated into clinical practice. The continued lack of impact on policy on the delivery of mental healthcare suggests that change may have to be community driven. Systemic service user advocacy groups could contribute critically to promoting authentic service user participation in the co-production of mental health services.

## Background

Facilitation of service user participation in the co-production of mental healthcare planning and service delivery is an integral component of contemporary mental health policy and clinical guidelines [1–3]. However, many service users continue to experience exclusion from the planning of their care [4]. Systematic reviews have reported that service users have requested more information, increased involvement in decision-making, and the provision of more substantive care choices [5–7]. Such calls have been sustained over time, which indicates an ongoing lack of policy effect on service delivery [2, 4].

In light of the importance mental health policy places on service user participation in service delivery, and the lack of impact such policies have on clinical practice, it is timely to synthesise the available evidence to identify key processes that influence servicer user involvement in mental healthcare. Such findings could potentially inform service delivery and assist in promoting service user participation in mental healthcare.

The objective of this meta-synthesis was to explore participation in mental health care from the perspective of both service users and service providers to elicit essential differences and similarities in their experiences. The specific objectives were to synthesise qualitative findings in this area and thereby articulate essential processes that enable service user participation in mental health care.

## Methods

The reporting of this meta-synthesis adheres to the ENTREQ guideline [8] and its conduct is based on Sandelowski and Barroso’s procedures, comprising (a) a systematic search strategy (b) critical appraisal of qualitative studies and (c) synthesis of findings [9]. These procedures were adopted as they provide a comprehensive framework to undertake a qualitative meta-synthesis, which when adhered to results in trustworthy and credible findings [10].

### Search strategy

**Fig. 1:**
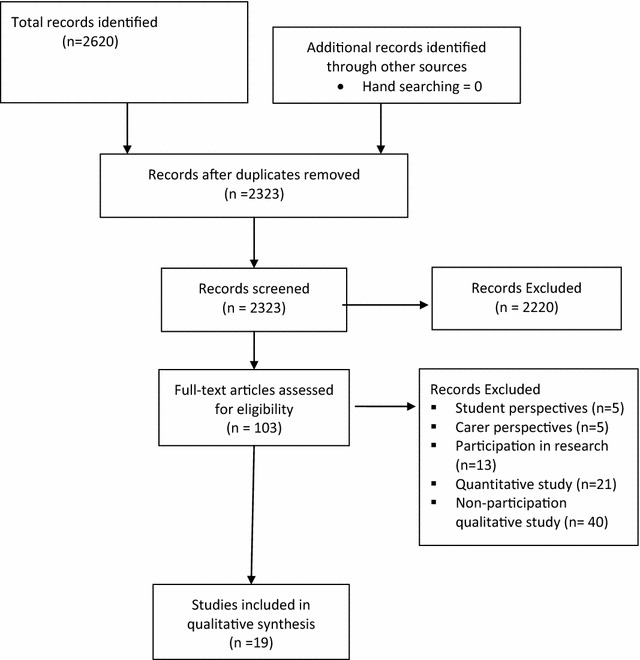
Implementation of search strategies and selection of studies

Figure 1 displays the search method and yield of studies. The search strategies were developed to identify English language, qualitative studies exploring participation in mental health care. PubMed, CINAHL, and PsycINFO were searched from 2000 to August 2015. We elected to limit the search to studies published from January 2000 to ensure that the studies’ findings reflected relatively current practice. For each database, a combination of subject headings and keywords were used with the combination modified as per each database’s controlled vocabulary (Appendix 1 presents the full search strategies). The titles and abstracts for all studies retrieved by the initial searches were screened by one of the authors to identify potentially relevant studies.

All appropriate studies were then reviewed against the inclusion criteria. Studies were eligible for inclusion if they were (a) studies detailing service users or service providers’ views about participation in mental healthcare (b) peer reviewed studies published in English between 2000 and 2015, and (c) studies used either qualitative methods or mixed methods. Studies were excluded if they: (a) detailed student or caregiver perspectives about service user involvement in mental healthcare; (b) focused only on service user participation in research; or (c) exclusively reported quantitative findings about service user involvement in mental healthcare.

### Search outcome

The search strategy yielded 2620 potentially relevant studies, of which 297 were duplicates. After title, abstract, and full text screening, 19 studies were included in the meta-synthesis. Table 1 displays the included studies, their purpose and method, classification of the findings, and overall critical appraisal score.

**Table 1:** Summary of the characteristics of the included studies

Source/country	Purpose	Method/data collection	CASP total
Bennetts et al. [23]/Australia	To explore what managers believe consumer participation to be, how they view the role of consumer consultants, and how they developed their understandings of consumer participation	Thematic analysis involving semi-structured interviews with 7 senior mental health managers working within an organization that had included consumer participation for many years	CASP = 20
Chong et al. [24]/Australia	To explore the perceptions of different healthcare professionals on shared decision-making and current interprofessional collaboration in mental healthcare	Thematic analysis involving interviews with 11 pharmacists, 7 nurses, 4 psychiatrists, 4 general practitioners, 2 social workers, 2 occupational therapists, and 1 psychologist	CASP = 18
Connor and Wilson [13]/United Kingdom	To obtain the views of a sample of users of mental health services regarding user involvement	Grounded theory study involving focus groups with 31 service users	CASP = 22
El Enany et al. [14]/United Kingdom	To explore the role of the user, and the basis of their ‘lay’ knowledge, in user involvement processes	Deductive reasoning involving semi-structured interviews with 35 health professionals and 28 service users	CASP = 18
Elstad and Eide [15]/Norway	To add to our understanding of user participation by exploring the experiences of users and professionals within a community mental health service	Thematic analysis involving interviews with 10 service users of community mental health centres	CASP = 24
Galon and Graor [16]/United States	To describe the social process of engagement in primary care treatment from the perspective of persons with severe and persistent mental illness	Grounded theory study involving semi-structured interviews with 32 service users	CASP = 21
Goodwin and Happell [25, 26]/Australia	To contribute to the body of knowledge regarding psychiatric or mental health nurses’ attitudes toward consumer and carer participation in mental health care	Content analysis involving focus groups with 30 psychiatric nurses	CASP = 16
Kidd et al. [17]/Australia	To explore the perceptions of consumer advocates and clinicians about the concept of consumer participation in mental health services and examine how consumer participation policy initiatives are enacted at a service delivery level	Thematic analysis involving semi-structured interviews with 8 health professionals and 2 consumer representatives, all of whom were members of a steering committee of mental health services	CASP = 19
Lammers and Happell [19]/Australia	To explore and discuss the perceptions of mental health service consumers regarding the reality of available opportunities for participation in the development, delivery and evaluation of mental health services	Thematic analysis involving in-depth interviews with 15 service users	CASP = 19
Lester et al. [18]/United Kingdom	To describe the views on, potential for, and types of patient involvement in primary care from the perspectives of primary care health professionals and patients with serious mental illness	Thematic analysis involving 18 focus groups with 45 service users, 39 general practitioners, and 8 practice nurses	CASP = 14
Lloyd [30]/United Kingdom	To identify the methods of empowerment used by mental health nurses	Thematic analysis involving semi-structured interviews with 10 mental health nurses	CASP = 23
Matthias et al. [28]/United States	To explore how consumers and providers make decisions in medication management consultations	Thematic analysis of directly observed medical consultation involving 4 providers, each of whom provided one consultation with 10 service users	CASP = 16
Petersen et al. [22]/Denmark	To gain insight into how the user’s understand and experience user involvement in mental health rehabilitation	Ethnographic study involving field observations and interviews with 12 service users	CASP = 24
Restall and Strutt [21]/Canada	To explore the perspectives of people who use mental health services on participation in mental health service planning and evaluation	Thematic analysis involving interviews and focus groups with 63 service users	CASP = 13
Rise et al. [31]/Norway	To investigate and compare service users’ and service providers’ own definitions of patient and public involvement and their implications	Grounded theory study involving semi-structured interviews with 20 patients, 13 public representatives and 44 health service providers/managers	CASP = 18
Rise et al. [32]/Norway	To investigate the experiences of professionals and service user representatives who took part in the implementation of a comprehensive development plan intended to enhance user involvement in a mental health hospital	Thematic analysis involving interviews with 5 service users and 13 health professionals. Ten meetings between service users and health professionals were also observed and notes were taken	CASP = 18
Robert [33]/United Kingdom	To explore the involvement of mental health service users in the redesign of in-patient mental health services	Thematic analysis involving 65 semi-structured exploratory interviews undertaken with project managers, team members, user representatives and other key informants and stakeholders	CASP = 16
Solbjor et al. [29]/Norway	To investigate mental health service users’ and providers’ views on patient participation during episodes of mental illness	Grounded theory study involving interviews with 17 patients, three service user representatives, 17 health professionals and 8 managers	CASP = 18
Summers [34]/United Kingdom	To explore psychiatrists’ views on active user involvement in the development of local NHS services	Thematic analysis involving semi-structured interviews with 14 psychiatrists	CASP = 16

### Critical appraisal

Before undertaking the meta-synthesis, one of the researchers evaluated each paper using eight questions contained in the Critical Appraisal Skills Program (CASP) checklist. We adopted a scoring method that had been previously used in several meta-syntheses [11]. For each of the eight CASP questions, one point was awarded when no, or scant, details were provided; two points were awarded when the issue had been addressed but not fully detailed; and three points were awarded when the issue was comprehensively addressed. For each article, the scores for the eight questions were summed, resulting in a maximum score of 24. The overall CASP score was provided as an indicator of the studies’ quality, but was not used to include or exclude studies from the synthesis.

### Data abstraction and synthesis

A preliminary set of 11 themes was developed by one researcher through line-by-line coding [12], which identified salient concepts in the studies included in this synthesis. These concepts were extracted into an excel spreadsheet. The extracted data took the form of either first order constructs that reflected the participants’ views as presented as excerpts in the included articles, or second order constructs that involved the interpretations or conclusions reported by the authors [9]. Two researchers then multiple coded the preliminary themes and used constant comparison to synthesise the initial dataset into a final set of six themes [12]. Any disagreement about the coding or synthesis of concepts was resolved through consensus.

## Results

The combined total of participants in the 19 studies included in this meta-synthesis was 662. Of these participants, 320 were clearly defined as service users, 16 as public representatives, and 220 as some type of health professional (nurses, general practitioners, psychiatrists, pharmacists, social workers, psychologists, occupational therapists, and mental health managers). The remaining 106 participants consisted of an unspecified combination of health professionals, managers, and user representatives. As can be seen from the composition of the participants in the included studies, this meta-synthesis reports diverse perspectives about what participation in mental health entails.

### Critical appraisal

The results of the critical appraisal are presented in Table 2. As can be seen, most of the included studies comprehensively reported details related to recruitment, data analysis, findings, and the value of the research. Alternatively, a minority of the included studies adequately addressed details regarding the justification of the research design and how the relationship between the researcher and participants may influence the findings.

**Table 2:** Results of the critical appraisal

	No/scant details (%)	Addressed but not fully detailed (%)	Comprehensively addressed (%)
Was the research design appropriate to address the aims of the research?	52.6	26.3	21.1
Was the recruitment strategy appropriate to the aims of the research?	0	15.8	84.2
Was the data collected in a way that addressed the research issue?	10.5	47.4	42.1
Has the relationship between researcher and participants been adequately considered?	73.7	5.3	21.1
Have ethical issues been taken into consideration?	26.3	36.8	36.8
Was the data analysis sufficiently rigorous?	15.8	26.3	57.9
Is there a clear statement of findings?	5.3	0	94.7
How valuable is the research?	5.3	5.3	89.5

### Meta-synthesis

The synthesis resulted in the identification of six principal themes, which articulate key processes that facilitate service user participation in mental healthcare. These themes included: exercising influence; tokenism; sharing knowledge; lacking capacity; respect; and empathy. The following sections present these themes in detail.

### Exercising influence

The ability to exercise influence was a core element of participation in almost all of the included studies [13–29]. For service users, exercising influence commonly related to making decisions about medication [13, 16–18], although it also extended to other issues such as selecting the menu or activities offered at in-patient facilities [15].

Service users often qualified their ability to exercise influence by noting that they did not desire absolute control, but instead wanted to share responsibility with health professionals in making decisions [16, 17, 19, 21]. Being able to influence decisions resulted in service users perceiving that health professionals thought that they were capable and credible, which contributed importantly to enhancing self-esteem [16, 17, 22]. However, service users said that health professionals frequently denied them the ability to influence decisions [13, 18, 22, 26]. As an example, one service user noted that:

“When I first went to him, he said “You should have medication”. But I didn’t want that. And he said he wouldn’t be able to treat me if I didn’t have medication. His way or no way, you know what I mean. That’s when I felt the control had been taken out of my hands [18].”

This lack in independence was a source of frustration and led to service users using covert strategies, such as withholding information, to reassert influence over decision-making [22, 23].

Health professionals perceived that service users’ views and needs should be acknowledged and that they should be informed of treatment decisions, but held disparate opinions about the extent to which service users should be involved in making decisions. In only one study was it noted that consumers should have the right to make decisions, regardless of the possible consequences [24]. For instance, one health professional stated that:

“If a person has the ability to refuse treatment, has the ability to consent in a reasonable way, yes absolutely we should respect it. Even if it means a poorer outcome for the person. That is their choice at the individual level [24].”

In general, though, health professionals indicated that their role was to enable service users to have some influence over decision-making, but should take control of decisions when they perceived that service users’ decisions were detrimental [15, 20, 24, 27, 29]. However, this begs the question: if health professionals allow service users to make decisions only when the decision reflects the health professional’s own view about the correct decision, do health professionals’ enable service users to make decisions or instead maintain the appearance of facilitating service user participation?

Health managers were aware of the health professionals’ apparent reluctance to fully enable service users to make decisions, noting that staff were more institutionalized than service users and were often unable to relinquish their authoritarian stance [14, 23]. However, this issue was only explicitly addressed in two studies and it remains unclear whether health managers generally perceived that health professionals maintained control over decision-making. Nonetheless, health managers stated service user consultants could potentially exercise a substantial degree of influence in changing the practice of health professionals [20, 23].

### Tokenism

Tokenism was a central theme of almost half of the studies included in this review, and it was closely related to the theme “exercising influence” since it revolves around maintaining the appearance of facilitating participation [14, 15, 17, 19–21, 23]. Surprisingly perhaps, even service users were complicit in enacting tokenistic behaviour [14, 19]. Health managers and health professionals would encourage particular service users to act as consultants when they knew that the service users would reflect their own positions [14, 20]. As one service user put it:

“I think a lot of healthcare professionals think (service user volunteers) are a nuisance unless they’re like me. People (health professionals and managers) look up to us (service user consultants) because we become one of them… you can do it you become one of them… they accept me on their level. They use me as a token a lot when they need a service user, in fact I’m probably doing the service user a disservice really because they use me, ‘oh we want a service user on this committee… we’ll get Adam [14]’”.

Consequently, the views of service user consultants were not necessarily representative of service users in general [15, 17]. Hence, the involvement of consumer consultants may sometimes only be a tokenistic demonstration of service user participation in mental health care. Some service user consultants explicitly recognized that they were inauthentically reflecting service users’ views, but justified it by noting that it was important for consumers to have a voice in committee decisions [14].

Even though service users were able to put forward their views during meetings about mental health services there was often no tangible outcome [15, 17, 19, 21]. As several studies noted, the notion of service user participation was commonly included as a mandatory element of mental health policy and managers were required to implement it throughout organisations [17, 19, 20]. However, the enactment of mandatory policy may have only been perfunctory, as reflected by this statement from a manager: “You’re nodding because you have to nod to show that you respect the consumer consultant, but really, do you believe it? No.” [23]. Finally, it should also be recognized that the commitment of policy makers did not always extend beyond rhetoric as no funding was provided to improve service user participation in mental health care [17, 25]. Essentially, the tokenistic actions of the health managers and health professionals was a type of collusion that was cloaked in rhetoric.

### Gaining knowledge

The importance of service users being sufficiently knowledgeable about mental healthcare was noted in almost all of the studies included in this review [13, 14, 16, 18–20, 22, 23, 25–29, 33, 35]. Becoming knowledgeable was seen as essential to enabling participation in mental health care [13, 20, 22, 26, 27, 33]. However, service users commonly stated that they lacked the ability to participate in decision-making either because the information provided was inadequate or health professionals used inaccessible jargon [13, 14, 16, 19, 33]. This was reflected in the following service user’s statement:

“I have heard a lot of cases where the other people on the Board of Management are speaking a language that those consumers could not possibly understand and therefore they can’t even give an opinion [19].”

Most service users wanted health professionals to educate them further about their condition and the available services [13, 16, 19, 22], although some service users stated that the onus was on themselves to take the initiative in becoming more knowledgeable [14].

Although all health professionals acknowledged the importance of ensuring that service users were capable of participating in decision-making, their commitment to this appeared to differ across professions. In general, nurses highlighted that treatment decisions should be based on a shared understanding, achieved through using accessible language and education [20, 26, 27]. Alternatively, physicians and psychiatrists seemed more likely to overuse jargon and less inclined to engage service users in decision-making, but it depended on the type of decision. If the decision was medication related, physicians and psychiatrists tended to openly discuss treatment options and concerns [28, 35]. As one health professional noted:

“It is an interaction between you and me. And I can’t cure you just like that with a pill… it’s about shared understanding and motivation and whether you agree with me or not. And whether you want to try what I think we ought to try. So it’s a lot of interaction and dialogue that leads somewhere [20].”

However, when service users asked questions about mental health or raised non-medication issues, physicians and psychiatrists generally responded in a cursory manner [35]. This lack in communication that physicians and psychiatrists displayed was also reported by nurses, who noted that they did not receive sufficiently detailed information about medication regimes, which in turn impaired communication between nurses and service users [26].

When health professionals discussed education issues, rarely did they broach what could be learnt from service users. In contrast, service users perceived that they were “experts in their own lives”, and therefore best understood mental health problems and could contribute significantly to improving mental health care [19, 20, 22]. Health managers typically echoed this view, stating that service users provided important perspectives on service delivery [14, 23]. The apparent failure of health professionals to recognize that both parties can benefit from education reinforces the often one-sided nature of participation that has been detailed in the previous themes in this review.

### Lacking capacity

Lacking the capacity to participate was a common theme, although it was only reported in a minority of the studies involving service users [15, 17, 29], but detailed in almost all studies eliciting the views of health professionals [17, 20, 24–27, 29, 33]. Service users typically acknowledged that decision-making should generally be left to health professionals or service user advocates during periods of severe psychosis [17, 19, 29]. Health professionals, though, did not mention advocates and health professionals often exerted control over decisions when service users were not in severe psychotic states but in their view lacked insight and were unable to make decisions in their own best interest [20, 24, 27, 29]. One health professional noted that:

“You need to try and get them on board as long as possible but when it comes to a point where the judgment is impaired, their reputation is at stake, I think we pass a barrier where the risks are now outweighing the benefit of allowing them to make these choices [24].”

This concept of “best interest” was frequently mentioned but remained nebulous and what it entailed was unclear. Again, it is evident that health professionals use various reasons to exclude service users from participation in mental health care.

### Empathy

Service users stated that empathy was an integral aspect of mental health care [13, 20]. As one service user noted: “a fundamental thing must be to be heard, seen, and valued. With that done I guess there are a million possible approaches. But that’s the essential thing [20]”. Empathy is clearly associated with participation because as the service users noted it involves being heard and receiving validation, followed by a tangible outcome that resulted from the interaction between the service user and health professional [13, 16, 17, 20]. Also, when service users experienced a lack of empathy from health professionals it could undermine cooperation and inhibit participation [29]. Health professionals also noted the importance of developing an empathetic relationship, especially as it provided service users with hope and optimism [25–27]. This position was captured in the following health professional’s statement:

“Often I just need to validate their experience because they’ve been very distressed, and if something’s positive, then you can give them a bit of energy to go away with, a bit of optimism and hope [25].”

### Respect

An important consequence of participation in mental health care was the sense of respect that service users experienced [16, 17, 20–22, 29]. One service user put it as: “where both parties feel respected and not overruled. Both must be allowed to say what they think and feel [20]”. As that statement indicated, this sense of respect was evoked when health professionals listened to service users and acted on their preferences for treatment, particularly as service users’ perceived that it signaled that they were capable and credible [16, 20, 22, 29]. In essence, then, respect meant turning rhetoric into meaningful action. When this occurred, service users noted that feeling respected enhanced recovery as it promoted independence [20, 22], which the health professionals also reflected: “this is the reason for us being here—helping them to feel competent to participate in the world” [15].

## Discussion

Our synthesis identifies critical transactional processes that are associated with service user participation in mental healthcare. Gaining knowledge was essential to enabling participation in mental healthcare, but it was useful only when service users were able to exercise influence over decision-making. Health professionals acknowledged the importance of including service users in decision-making, but rarely conceded that service users should have control over decisions. Although health professionals indicated that service users should participate in service delivery, it was often tokenistic and service user preferences were typically only incorporated when they accorded with health professionals’ views about appropriate treatment. Service users stated that empathy contributed importantly to promoting participation, which health professionals also acknowledged. Finally, an important outcome of genuine participation in mental healthcare was the sense of respect service users experienced.

Some of the key concerns that emerged from this synthesis were the lack of service user involvement and enactment of tokenism, whereby service users were involved in consultation without subsequent collaboration, or particular service users were included in discussions because their views aligned with health professionals’ perspectives. Such practices also commonly occur in medical fields other than mental health, which suggests that tokenism results from systemic cultural attitudes [36–38]. Fostering more inclusive approaches to service user involvement will require additional training of mental health professionals, which then needs to be embedded in clinical contexts where authentic partnership is standard practice.

This shift in the imbalance of power will probably not occur without the involvement of systemic service user advocacy groups in determining mental health care priorities [39, 40]. Hence, it is critical that policy stakeholders include such advocacy groups in planning mental health services. In addition, cultural change requires time, and more immediate solutions are therefore required to foster service user participation in decision-making. As such, the importance of individual advocacy needs to be highlighted, as research has shown that health professionals are more accommodating of service user preferences when advocates attend consultations [41, 42].

Mental health service providers often hold stigmatising beliefs about service users’ lack of capacity to make informed decisions about their care [43, 44]. Such beliefs were captured in two of the themes reported in this synthesis: “exercising influence” and “lacking capacity”. The presence of these beliefs draws attention to the need to deliver interventions that reduce the extent to which service providers stigmatise service users. Two interventions that may be useful to reduce service providers’ erroneous attitudes involve education and contact [44]. The educational approach counters stereotypes through comparing the myths of mental illness with facts [45]. Contact interventions also seek to mitigate stereotypes, but achieve it through exposing service providers to high functioning individuals with a mental illness [45]. The implementation of such interventions could be considered to promote affirming behaviour among mental health service providers towards service users.

Almost all of the studies included in this synthesis drew attention to the importance of improving service users understanding of care options. However, better informed service users are more likely to question the authority and expertise of health professionals, which tends to result in service users being labeled as “difficult patients” who may consequently receive substandard care [46–51]. It is unclear if health professionals are aware of the inconsistency between their stated position and lack of acceptance of its inevitable outcome, but it is an issue that warrants further examination as such dissonance will probably impair the recovery of service users.

It seems incongruous that health professionals fail to recognise the contribution service users can make in educating staff about the importance of participative relationships in facilitating recovery. In particular, a relationship built on genuine participation provides a basis for more accurate assessments of recovery and relapse, from which appropriately tailored interventions can be implemented.

Finally, the results of the critical appraisal provide some guidance for improvement in the conduct of further qualitative studies that explore service user participation in mental health care. Of particular note was the lack of details that were reported regarding the relationship between the researcher and participants. Such relationships are important to understand since it can influence the participants’ responses, or the manner in which the researcher interprets the data [12]. Full disclosure of the relationship between the participants and researcher enhances the credibility of the findings [12]. The other reporting issue that was inadequately detailed in the majority of the included studies was information concerning the rationale behind selecting a particular research design to address the aims of the study. The provision of these details facilitates an understanding of how the theoretical framework shaped the investigation of the research aims [12].

### Limitations

Our presentation of the qualitative meta-synthesis is but one possible interpretation of the included studies findings. Examining patterns throughout diverse participant groups typically omits detailed interrogation of the complex experiences within each group. Nonetheless, our synthesis was derived from the views of service users, health professionals, and managers, and hence includes the perspectives of all important stakeholders. We therefore believe our findings capture the essential processes influencing participation in mental healthcare. However, all of the studies included in this synthesis were conducted in high-income, developed countries. It may be the case that in developing countries involvement in mental healthcare may differ from the manner in which it has been conceptualised in this study, particularly as there might be substantial variation in cultural practices between developed and developing countries. Finally, the search strategy was undertaken in PsycINFO, PubMed, and CINHAL, and hence pertinent studies indexed only in other databases may have been excluded from this review. In addition, searches were not undertaken of the non-indexed literature, which may have also led to the omission of relevant studies.

## Conclusion

The findings of this meta-synthesis demonstrate that service user participation in mental healthcare remains a policy aspiration, which generally has not been translated into clinical practice. The continued lack of impact of policy on the delivery of mental healthcare suggests that change may have to be community driven. Systemic service user advocacy groups could contribute critically to promoting authentic service user participation in the co-production of mental health services. Policy stakeholders could also consider placing service users as leaders in key positions throughout mental health services, which may help in shifting the culture of mental health professionals towards a more recovery focused approach.

## Appendix 1: Electronic search strategies

### Medline search strategy

(involve* OR participat* OR facilitat* OR engage* empower* OR collaborat* Patient Participation (MH) OR Community Participation (MH) OR Decision Making (MH)) **AND** (consumer OR “service user” OR survivor OR patient OR client OR people) **AND** (Mental Health (MH) OR Mental Disorders (MH) OR “mental health” OR “mental illness” OR schizophrenia OR bipolar OR psychosis) **AND** Qualitative Research (MH) OR qualitative OR “mixed methods” OR “action research” OR Focus Groups (MH))

### CINHAL search strategy

(MH Consumer Participation+ OR MH Decision Making+ OR MH Empowerment+ OR TX decision making OR TX involve OR TX involvement OR TX participate OR TX participation OR TX engage OR TX engagement OR TX facilitate OR TX facilitation OR TX empowerment OR TX collaborate OR TX collaboration) **AND** (MH Mental Health+ OR MH Mental Illness OR MH Attitude to Mental Illness OR OR “mental health” OR “mental illness” OR schizophrenia OR bipolar OR psychosis**) AND** (MH Qualitative Studies+ OR MH Action Research+ OR TX qualitative OR TX mixed methods OR TX action research OR TX focus group) **AND** (TX consumer OR TX service user OR TX client OR TX patient OR TX survivor OR TX patient OR TX people)

### PsycINFO search strategy

(EXACT “client participation” OR EXACT “decision making” OR EXACT “involvement” OR EXACT “empowerment” OR involve* OR participat* OR facilitat* OR engage* OR empower* OR collaborat*) **AND** (EXACT “mental health” OR EXACT “chronic mental illness” OR EXACT “mental illness (attitudes towards)” OR “mental health” OR “mental illness” OR schizophrenia OR bipolar OR psychosis**) AND** (consumer OR “service user” OR survivor OR patient OR client OR people) **AND** (EXACT “qualitative methods” OR EXACT “action research” OR qualitative OR “mixed methods” OR “action research”)
